# MOF derived core-shell CuO/C with temperature-controlled oxygen-vacancy for real time analysis of glucose

**DOI:** 10.1186/s12951-022-01715-z

**Published:** 2022-12-01

**Authors:** Chen Zhao, Xiaoying Tang, Jinge Zhao, Jie Cao, Zhenqi Jiang, Jieling Qin

**Affiliations:** 1 Shanghai Tenth People’s Hospital, School of Medicine, Tongji University Cancer Center, Tongji University, Shanghai, 200092 China; 2 School of Medical Technology, School of Life Science, Beijing Institute of Technology, Beijing, 100081 China; 3 Key Laboratory of Medical Molecule Science and Pharmaceutics Engineering, Ministry of Industry and Information Technology, Key Laboratory of Cluster Science of Ministry of Education, Beijing Key laboratory of Photoelectronic/Electro-photonic Conversion Materials, School of Chemistry and Chemical Engineering, Beijing Institute of Technology, Beijing, 100081 People’s Republic of China

**Keywords:** Metal-organic frameworks, High-temperature pyrolysis, Oxygen-vacancy, Direct glucose sensing, Non-enzymatic sensor

## Abstract

Introducing oxygen-vacancy into the surface of the non-enzymatic sensor is supposed to be an effective way to improve inherently low catalytic activity and specificity of non-enzymatic sensors. In this work, CuO/C was synthesized at different temperatures using metal-organic frameworks as sacrificial templates to receive additional content of oxygen-vacancy. The product with the highest oxygen vacancy was found at 400 °C (named CuO/C-400 °C), which increased catalytically active sites and enhanced the charge-transfer efficiency. The sensing performance was afterward explored by amperometry under an optimal applied potential at 0.5 V (vs. SCE), presenting a broad detection range from 5.0 µM to 25.325 mM (R^2^ = 0.9998) with a sensitivity of 244.71 µA mM^− 1^ cm^− 2^, and a detection limit of 1 µM. Furthermore, the reliability and selectivity of CuO/C-400 °C sensors were extensively explored in the presence of artificial serum/saliva samples with gradient glucose concentrations. The human blood samples were also detected with high recoveries compared with the clinical Hexokinase method. Hence, the prepared CuO/C-400 °C sensor with a broad detection range and high selectivity can be applied for the diabetes diagnosis ex vivo without further dilution for real-time analysis in practical applications.

**Supplementary Information:**

The online version contains supplementary material available at 10.1186/s12951-022-01715-z.

## Introduction

Diabetes is a chronic disease that occurs when insulin is neither well-produced nor effectively utilized. Over 4.2 million people died from diabetes in 2019 [1], and this number is projected to constantly increase to about 700 million by 2040 s, according to the estimation of the International Diabetes Federation (IDF) [2]. Precise control of blood glucose levels in daily life can not only reduce the symptom and increase the survival rates of diabetes, but also prevent or delay long-term, serious health problems, such as heart disease, vision loss, and kidney disease, so the development of glucose monitoring devices in real-time is necessary [3]. Although commercial glucose oxidase-based glucose meters have been widely used, the sensing performance is easily interfered with by the external storage and transport conditions, which hinders their applications in glucose determination [4]. Non-enzymatic glucose sensors (such as metal oxides), in that case, have attracted much attention for their high chemical stability and easy operation. Nevertheless, compared with glucose oxidase enzymes, the catalytic activity and specificity of non-enzymatic materials are usually lower, resulting in a poorer sensing performance [5–7].

CuO, which is expected to be an attractive non-enzymatic sensor for its p-type semiconductor with a narrow bandgap of 1.2 eV, has been widely explored [8, 9], especially for non-enzymatic glucose sensors [10–12]. Still, the detection range of most as-prepared sensors is 0–5 mM which is narrower than the glucose in human blood (4–7 mM for healthy people and ≥ 9 mM for diabetic patients) due to the weak conductivity of CuO [13, 14]. While introducing oxygen vacancies by adjusting defect structures and electronic states, such as heating the material in a reductive atmosphere, and doping nonmetals (halogen, etc.) to replace lattice oxygen [15, 16], has been suggested to improve the conductivity of CuO [17, 18]. However, these methods often require secondary heating, and the reductive atmosphere is prone to make crystal agglomerate and collapse. Thus it is urgent to develop a technique to increase the oxygen-vacancy content in CuO without secondary heating.

Metal-organic frameworks (MOFs) are composed of metal ions and organic links through coordination bonds with regular pores. These have been widely applied in biomedicine, such as sensors, bioimaging materials, and drug delivery agents [19–22]. Recently, metal oxide/C-derived MOFs have attracted much attention in the electrochemical field for designing appropriate structures with plenty of active species [23]. For example, thermal treatment can reduce the reaction time with no post-treatment, whilst the product keeps the morphology of the initial MOF with a large surface area [24, 25]. More importantly, such treatment allows more atomically active sites to be exposed, resulting in better MOF performance. Recently, many groups found that oxygen-vacancy in MOF-derived materials can enhance electrochemical performance [26]. Hence, exploring MOF-derived CuO with more atoms active-sites and oxygen-vacancy will be a promising way to achieve a more comprehensive linear range for glucose sensing. However, the change of oxygen-vacancy under different processing temperatures is seldom reported for the MOF-derived sensors.

Given the above-mentioned advantages and consideration, in this study, the Cu-MOF was synthesized through the coordination of copper ions and homophenolic acid, followed by the calcination in the furnace at different temperatures (350 ^o^C, 400 ^o^C, and 450 ^o^C) to receive the CuO/C core-shell nanoparticles with oxygen vacancy. The thermal treatment of Cu-MOF under different temperature were further found to create active sites and increase the oxygen-vacancy for the electrocatalytic oxidation of glucose. Compared with CuO/C-350^o^C and CuO/C-450^o^C, the developed CuO/C-400 °C has the most oxygen-vacancy and the highest response toward glucose oxidation in primary media. The wide detection range of glucose was explored using CuO/C-400 °C modified glassy carbon electrode (GCE) by amperometry under an optimal applied potential at 0.5 V due to the oxygen-vacancy and adsorbed hydroxyl ions. The sensing performance was then verified in artificial serum/saliva and human blood sample in real time analysis with remarkable reproducibility.

## Experimental

### The synthesis of CuO/C at different temperatures

Cu-MOF was first synthesized at room temperature (RT) by liquid phase method. In detail, 3 mmol Cu(NO_3_)_2_·6H_2_O was dissolved in 25 mL deionized water, while 2 mmol 1,3,5-benzenetricarboxylic acid and 6 mmol triethylamine were mixed in 25 mL ethanol. Then two solution was mixed under stirring and kept for 24 h at RT. The final blue powder was received after the purification with ethanol and dry at 60 ^o^C for 24 h.

For the preparation of CuO/C nanoparticles, the synthesized Cu-MOF (100 mg) was afterward placed in a porcelain boat and calcined in a muffle furnace with the increased temperature at a rate of 1 ^o^C/min. After reaching the specified temperature, the temperature was kept constant for 2 h and then cooled down to RT. The final samples were black and named CuO/C-X^o^C (X = 350, 400, 450), depending on the calcination temperature.

### The preparation of the working electrode

0.3 μm and 0.05 μm alumina slurries were used sequentially to polish the glassy carbon electrodes (GCE; diameter, 3 mm), followed by the immersion in ethanol and ultrapure water in sequence for ultrasonic cleaning. For the preparation of the CuO/C-X ^o^C (X = 350, 400, 450) based working electrode, 2 mg of prepared CuO/C-X ^o^C powder was dispersed in 1 ml of ultrapure water under ultrasound for 30 min. Then 5 µL of the CuO/C-X ^o^C dispersion was directly dropped onto the surface of the pre-treated GCE and dried under 30 ^o^C. The GCE /CuO/C-X ^o^C electrode was obtained by repeating the drip - drying process for four times.

### Real samples detection

To further study the practical application of the sensor, 5 µL of the CuO/C-400 °C dispersant was dropwise added to the working electrode area of ​​the electrode (diameter: 3 mm) through the drip-drying process four times. The simulated serum sample was prepared using PBS containing 10% FBS, while simulated saliva was received from Chuangfeng Technology Company (Dongguan, China). The human serum samples (2 diabetics and 2 healthy) were kindly donated by Anzhen Hospital (Beijing, China) and tested using by Hexokinase method (Roche COBAS INTEGRA 800 automatic biochemical analyzer). The performance of the synthesized biosensor in complex biological samples containing different concentrations of glucose was investigated by amperometry under an applied voltage of 0.5 V.

## Results and discussion

### Synthesis and characterization of Cu-MOF

After the successful preparation of the Cu-MOF, the morphology and properties were examined by commonly used material characterization methods, including transmission electron microscope (TEM), EDS mapping, X-ray diffractometer (XRD), X-ray photoelectron spectroscopy (XPS), etc. TEM results showed that the prepared Cu-MOF was nanoparticles with the size of 50–80 nm, and energy dispersive spectrometer (EDS)-mapping represented that the synthesized nanoparticles were mainly composed of Cu, C, and O with uniform dispersion (Additional file 1: Fig. S1). The powder XRD patterns of the as-prepared Cu-MOF (Additional file 1: Fig. S2) further identified a good crystalline state that was in line with the previous literature [27], indicating the successful synthesis of Cu-MOF. Meanwhile, the high purity and good crystal quality of the Cu-MOF can be further confirmed by the observation of sharp and intense diffraction peaks. To further confirm the element and the chemical state, the prepared Cu-MOF was tested by XPS (Additional file 1: Figs. S3 and S4), revealing the presence of Cu, C, and O without other elemental contaminants. Furthermore, the high-resolution spectra of XPS can also confirmed the successful preparation of Cu-MOF containing Copper ion (II) and 1,3,5-benzenetricarboxylic acid.

In addition, the heat treatment mechanism of Cu-MOF under air was simulated by means of thermogravimetric-differential thermal analysis (TG-DTA) between 25 ^o^C and 600 ^o^C (Additional file 1: Fig. S5) with two obvious weight loss steps. The first mass loss, with 30.47 *wt*% from 25 to 296 ^o^C, relating to the removal of water and other molecules for negative of DTA. While the weight loss of 42.36 *wt*% from 296 ^o^C to 350 ^o^C indicated framework of Cu-MOF began to collapse at this stage. After 350 ^o^C, there was no noticeable weight loss could be observed, representing that the Cu-MOF was decomposed entirely and totally converted to CuO directly. Hence the 350 ^o^C, 400 ^o^C, and 450 ^o^C were selected to calcine Cu-MOF in this study to explore the changes of oxygen-vacancy content caused by thermal treatment temperature.

### Synthesis and characterization of CuO/C at different temperature

**Fig. 1:**
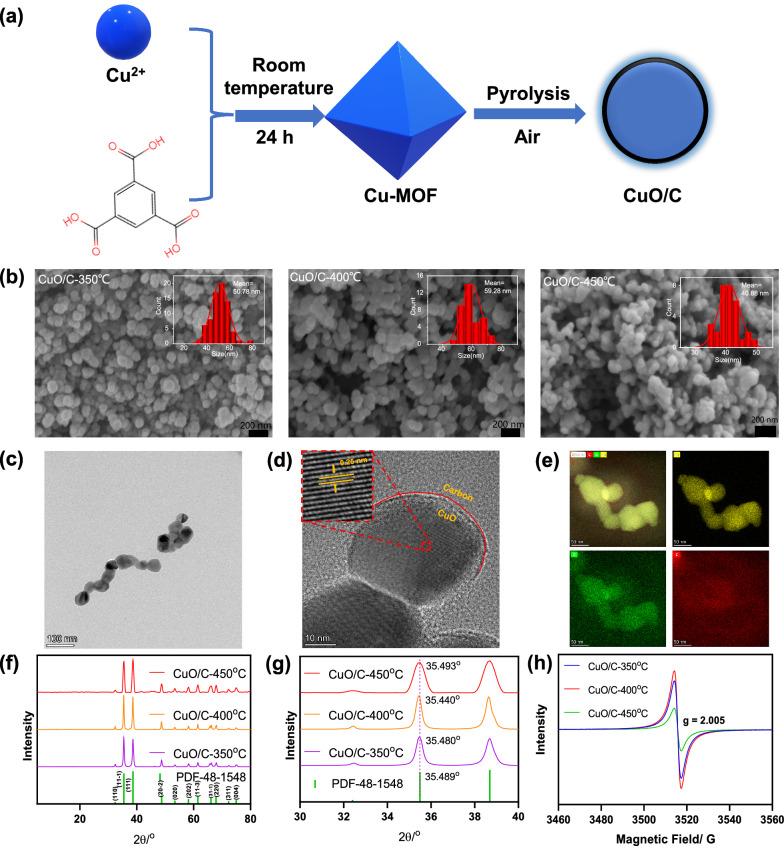
**a** Scheme illustration of the synthesis of CuO/C; **b** SEM images of CuO/C-350^o^C, CuO/C-400 °C and CuO/C-450 °C, the inset is the particle size statistics; **c** TEM, **d** HR-TEM and **e** Cu, O, C element mapping of CuO/C-400 °C; **f** XRD patterns of CuO/C-350 °C, CuO/C-400 °C, CuO/C-450 °C and the standard PDF of CuO; **g** XRD patterns for the 2Ɵ from 30˚ to 40˚; **h** EPR spectra of CuO/C-350 °C, CuO/C-400 °C and CuO/C-450 °C

The samples obtained after thermal treatment under air at different temperatures (350 ^o^C, 400 ^o^C, and 450 ^o^C) were named as CuO/C-350 ^o^C, CuO/C-400 ^o^C and CuO/C-450 ^o^C, respectively (Fig. 1a). In addition, a commercial CuO with a size of 40 nm was bought for comparison. The prepared samples at different temperatures and commercial CuO were first observed by scanning electron microscope (SEM). As shown in the SEM images (Fig. 1b and S6), the average size of prepared samples were calculated to be 50.78, 59.28, 40.88, and 44.95 nm for CuO/C-350 ^o^C, CuO/C-400 ^o^C, CuO/C-450 ^o^C, and commercial CuO, respectively. TEM, HR-TEM together with the mapping were used to further confirm the detail morphology of the prepared samples (Fig. 1c–e). Similar with the results from SEM, the size of CuO/C-400 ^o^C in TEM image was about 60 nm. Moreover, an amorphous carbon layer was observed to wrap on the surface of copper oxide, forming a core-shell structure of CuO/C by HR-TEM in Fig. 1d. Further well-defined lattice fringes with the d-spacing of the lattice fringes were measured to be 0.25 nm attributing to the (111) reflections of monoclinic CuO. Meanwhile, the EDS (Additional file 1: Fig. S7) and EDS-mapping analysis (Fig. 1e) were confirmed that the architecture of CuO/C-400^o^C contained Cu and O elements distributing homogenously in the entire architecture of CuO/C-400 ^o^C.

The crystal structures of the prepared samples and commercial CuO were also characterized by XRD (Fig. 1f and Additional file 1: Fig. S8). All the diffraction peaks can be indexed to the monoclinic-phase of CuO (JCPDS 48-1548). Specifically, the peaks with 2Ɵ of 32.406˚, 35.489˚, 38.694˚, 48.841˚, 53.403˚, 58.194˚, 61.498˚, 66.101˚, 67.909˚, 72.301˚, and 74.998˚ matched the crystal surface of monoclinic CuO without any signal from copper acetate or other precursor compounds, indicating the production of high purity single-phase CuO. Furthermore, the results of different samples with 2Ɵ from 30˚ to 40˚ were identified in a narrow range in Fig. 1g. As the under bound orbital of atom located on the nonbonding orbital of the transition metal due to the extra electrons generated with the oxygen-vacancy, causing the peaks of the crystal planes shift to lower angles. The tested angle of (111) was 35.48˚, 35.44˚, 35.493˚, and 35.579˚ for CuO/C-350 ^o^C, CuO/C-400 °C, CuO/C-450 ^o^C, and commercial CuO, respectively, demonstrating more oxygen-vacancies of CuO/C-400 ^o^C. In addition, the electron paramagnetic resonance (EPR) spectrum (Fig. 1h), which was proved to be an effective tool for manifesting the presence of atomic vacancy [28], was used to examine the formation of oxygen vacancies in the samples. All the samples displayed a symmetrical EPR signal at g = 2.005. Compared with the calcination at other temperatures, the signal strength of CuO/C-400 ^o^C was the strongest, indicating the highest content of oxygen vacancies. Herein, the CuO/C-400 °C might have the most oxygen-vacancy among all samples, indicating a potentially good electrochemical activity.

**Fig. 2:**
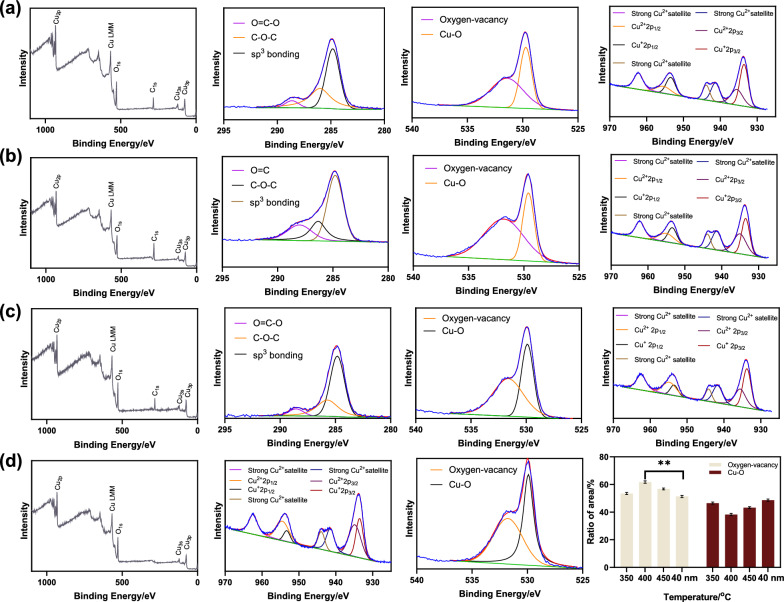
XPS spectra of **a** CuO/C-350^o^C, **b** CuO/C-400 °C, and **c** CuO/C-450 °C: survey scan, C_1 s_, O_1 s_ and Cu_2p_; **d** Survey scan, O_1 s_ and Cu_2p_ for commercial CuO (40 nm); **e** The ratio of oxygen-vacancy and Cu-O for CuO/C-350 °C, CuO/C-400 °C, CuO/C-450 °C and commercial CuO. (MEAN ± SD, n = 3, *p < 0.05)

To further investigate the surface element chemical states and oxygen-vacancy, the prepared CuO samples and commercial CuO were subjected to XPS testing. The peaks of C, O, and Cu could be observed in all prepared samples (Fig. 2a–c), whilst only Cu and O existed in commercial CuO (Fig. 2d). Moreover, the binding energy of Cu for all samples were similar, which indicated the Cu oxidation state with no noticeable difference. The spectra of C_1 s_ for the prepared samples showed there were a carbon layer with sp^3^ bonding on the surface which was consistent with the result of TEM images. For the high-resolution spectra of O_1 s_, there were two O_1 s_ surface peaks that could be fitted by two bands. The band with lower binding energy was ascribed to the lattice oxygen (Cu-O) of the CuO crystal lattice, corresponding to 529.7 eV, 529.6 eV, 529.9 eV, and 529.9 eV of CuO/C-350 °C, CuO/C-400 °C, CuO/C-450 °C, and commercial CuO, respectively. A shoulder band with higher binding energy was ascribed to the adsorbed oxygen or oxygen in hydroxyl-like groups on the surface of CuO (denoted as oxygen-vacancy). The band of oxygen-vacancy was related to the bands at 531.4 eV, 531.7 eV, 531.6 eV, and 531.8 eV of CuO/C-350^o^C, CuO/C-400^o^C, CuO/C-450 °C, and commercial CuO, respectively. Peak areas were used to calculate the relative content of different elemental states of O on the surface. The ratio of oxygen-vacancy to Cu-O was 1.15, 1.62, 1.31, and 1.05 for CuO/C-350 °C, CuO/C-400 °C, CuO/C-450 °C, and commercial CuO, respectively, indicating that CuO/C-400 °C had the highest oxygen-vacancy. The possible mechanism was that the increase of temperature might cause a carbon-mediated local reduction reaction at the surface of CuO/C, bringing an improvement in oxygen vacancies without disrupting the lattice. However, excessive temperature would lead to the structure collapse to reduce the oxygen-vacancy [29]. The experiment showed that with the increase of temperature (from 350 to 400 °C), oxygen dissociation is caused, leading to the generation of more oxygen vacancy. As the temperature further increased (400 to 450 °C), the structure collapsed, finally deducing the oxygen vacancy of CuO/C. The TG-DTA results indicated that the temperature increase did not lead to further loss of the mass or the collapse of the structure. However, the XPS results demonstrated that the change of temperature would lead to the oxygen-vacancy content change, together with the higher density of the surface defects, the surface adsorption sites, and the catalytic activity. Hence, the CuO/C-400 °C was expected to show good electrocatalytic ability toward glucose oxidation.

### Electrocatalytic performance of the obtained electrodes for glucose detection

**Fig. 3:**
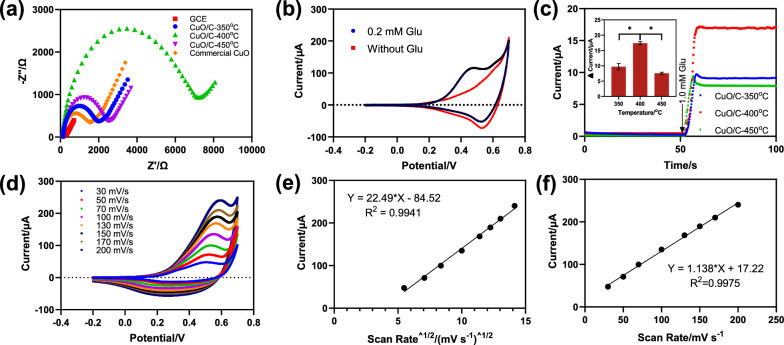
**a** Nyquist plots of the CuO/C-350^o^C, CuO/C-400 °C, CuO/C-450 °C and commercial CuO in 0.1 M KCl electrolyte containing 5 mM Fe(CN)_6_^3− /4−^ and an applied AC frequency range of 0.1–10^5^ Hz at 0.2 V (vs. Ag/AgCl) with an amplitude of 5 mV; **b** Cyclic voltammograms of the CuO/C-400 °C electrodes in 0.1 M NaOH with/without 0.2 mM glucose at a scan rate of 100 mV·s^− 1^; **c** Amperometric i-t response of the CuO/C-350 °C, CuO/C-400 °C and CuO/C-450 °C electrodes in 0.1 M NaOH at 0.5 V (vs. SCE) with stirring, insert is the response current density of 1.0 mM glucose on the CuO/C-350 °C, CuO/C-400 °C and CuO/C-450 °C electrodes derived from Fig. 3c (MEAN ± SD, n = 3, *p < 0.05); **d** CV curves of CuO/C-400 °C in 0.5 mM K_3_Fe(CN)_6_/0.1 M KCl electrolyte at different scan rate and (**e**), **f** the corresponding fitting curves

Electrochemical Impedance Spectroscopy (EIS) was used to analyze the mass transfer characteristics and the charge of components in sensors. Figure 3a showed the Nyquist plot obtained for the GCE, GCE/CuO, GCE/CuO/C-350 °C, GCE/CuO/C-400 °C, GCE/CuO/C-450 °C electrodes in 0.1 M KCl containing 5 mM Fe(CN)_6_^3− /4−^ with frequency from 0.1 to 10^5^ Hz at 0.2 V. The CuO electrode showed the smallest resistance value (Rct) was about 1544 Ω, while the Rct of GCE/CuO/C-350^o^C, GCE/CuO/C-400 °C, and GCE/CuO/C-450^o^C were 2003 Ω, 7244 Ω, and 2515 Ω, respectively, owning to the increase of surface oxygen vacancies increase can destroy the crystal structure in the nanoparticles, resulting in an increased conductivity of the sensors.

The CV curves were used to evaluate the performance of the prepared GCE/CuO/C-X^o^C (X = 350, 400, 450) sensors for catalyzing glucose. All electrodes were tested in 0.1 M NaOH solution with or without 0.2 mM glucose. As shown in Fig. 3b, the CV curves of the GCE/CuO/C-400 °C electrode showed a distinct oxidation peak in glucose, while no oxidation peak was observed without glucose. The catalytic oxidation ability of the other three materials was also tested under the same conditions (Additional file 1: Fig. S9). The largest catalytic oxidation current of the GCE/CuO/C-400 °C electrode indicated that the CuO/C-400 °C had the strongest catalytic oxidation capacity for glucose in comparison, which was related to the highest oxygen vacancy of the CuO/C-400 °C and can not only increase the charge transfer efficiency but also enhance the interaction between oxygen-containing species and the metal oxide surface effectively. The 0.5 V was selected as the applied potential for chronoamperometry detection as it had the highest response with the gradual addition of glucose under different potentials and enough driving force for the glucose oxidation reaction. The amperometric curves of different electrodes were carried out under 0.5 V with an addition of 1.0 mM glucose. The current responses of GCE/CuO/C-400 °C, GCE/CuO/C-350 °C, and GCE/CuO/C-450 °C to glucose were 17, 10, and 7 µA, respectively (Fig. 3c). It proved that the catalytic ability of the CuO/C-400^o^C material was significantly higher than that of the other two materials. Figure 3d showed the CVs at different scan rates for GCE/CuO/C-400 °C in electrolytes contained 0.2 mM glucose. In the range of 30 mV/s to 200 mV/s, the current increased with the aggrandizement of the scan rate. As shown in Fig. 3e, f, and Additional file 1: Fig. S10, the oxidation current had a linear relationship with the scan rate and the square root of the scan rate, indicating the co-existence of surface confinement and diffusion control in the CuO/C-X °C [30–32]. Moreover, as the slopes of the regression equation of GCE/CuO/C-350^o^C and GCE/CuO/C-450 ^o^C were significantly lower than that of GCE/CuO/C-450 ^o^C, which was related to the catalytic performance of the material.

**Fig. 4:**
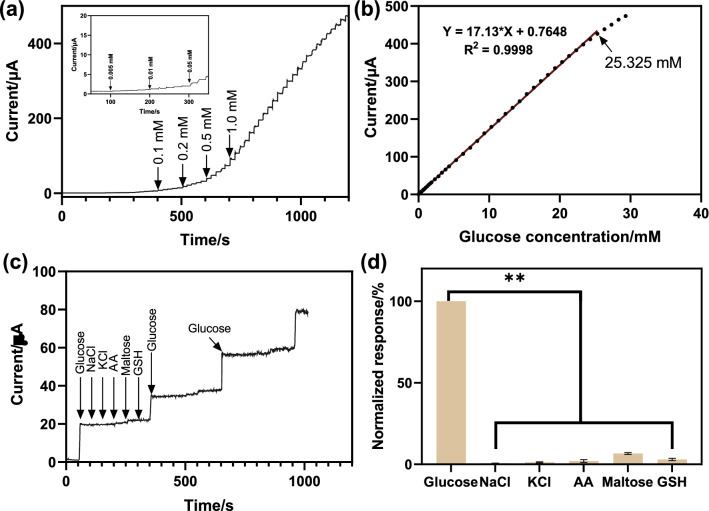
**a** Amperometric responses of CuO/C-400 °C in 0.1 M NaOH upon consecutive addition of glucose at 0.5 V (vs. Ag/AgCl); **b** the corresponding calibration curves of the CuO/C-400 °C for the glucose detection; **c**, **d** influence of interfering substances (0.1 mM NaCl, 0.1 mM KCl, 0.1 mM AA, 0.1 mM maltose and 0.1 mM GSH) on the amperometric response to glucose by CuO/C-400 °C sensor (MEAN ± SD, n = 3, **p < 0.01)

Figure 4a, b showed the i-t curve of GCE/CuO/C-400 °C along with the corresponding linear plots of the calibration curve. According to the results, the electrode exhibited a rapid response with the addition of glucose, indicating CuO/C-400 °C has high catalytic activity. The sensitivity and linear range of these modified electrodes could be obtained from calibration curves. Based on the catalytic ability of CuO/C, the electrode showed a prominent characteristic that the current response value gradually increases with the increase of glucose concentration. In addition, the concentration of oxygen vacancies on the surface of the CuO/C was controlled by changing the calcination temperature during the calcination process, and the catalytic ability of CuO/C-X °C (X = 350, 400, 450) to glucose was different, which in turn leaded to different sensitivity among GCE/CuO/C-X ^o^C (X = 350, 400, 450) electrodes. Among them, the GCE/CuO/C-400 °C electrode exhibited the highest sensitivity at about 244.71 µA mM^− 1^ cm^− 2^, while the sensitivities of GCE/CuO/C-350 ^o^C and GCE/CuO/C-450 °C electrodes were 140.69 and 79.06 µA mM^− 1^ cm^− 2^, respectively. Furthermore, the linear correlation between the response current (µA) and glucose concentration (mM) of the GCE/CuO/C-400 °C electrode was y = 17.13x + 0.7648 (R^2^ = 0.9998) with the linear range from 5.0 µM to 25.325 mM, and the limit of detection (LOD) of 1.0 µM (S/N = 3). Compared with other reported CuO-based non-enzymatic sensor in Table 1, our prepared CuO/C-400 ^o^C with abundant oxygen-vacancy through a simple preparation process without secondary heating. The MOF derived material offers larger surface area for more activated species and oxygen-vacancy leading to the enhanced charge-transfer efficiency. Furthermore, the thin carbon layer on the surface of the copper oxide during the formation process may facilitate the electron transfer, reduce the physical changes on the surface of CuO during the catalytic process, so as to better maintain the detection activity and repeatability of biosensor. Herein, the GCE/CuO/C-400 °C electrode showed remarkable electrocatalytic activity toward glucose oxidation with a wider detection range than other reported CuO-based nonenzymatic glucose probes.

**Table 1:** Comparison between the electrodes in this work and other Cu-based glucose sensor

Electrode	Type of sample	Sensitivity (µA mM^− 1^ cm^− 2^)	Linear range (mM)			Detection limit (µM)		References	
			From	To					
Copper–graphene nano–composite	Human serum sample	11.3	0.9	11		1		[33]	
Electrochemically reduced graphene oxide (ERGO)/Cu/GCE	Human blood sample	445	0.00014	5.091		0.049		[34]	
CuO NWs/SWCNTs/ GCE	Human serum diluted 20 times by 50 mM NaOH	761.5	0.034	2.67		0.0456		[35]	
Cu@C composite nanotube array	0.5 mM glucose in NaOH	1200	0.001	0.06		1		[36]	
3D Cu@Cu_2_O aerogels 0.1 M NaOH)	Human serum sample	194.88	0.001	17.12		0.6		[37]	
Cu_2_O microcrystals	Glucose solution	97	0	14.3		0.33		[38]	
Cu–MOF	Glucose solution	89	0.00006	5		0.0105		[39]	
CuO–350–NA/GCE	Fusayama’s artificial saliva	18.061	0	6.535		0.15		[40]	
Cu–MOF/MWNTs	Human serum sample	3878	0.0005	2.34		0.4		[41]	
Cu_2_O/AlOOH/rGO	Glucose solution	155.1	0.005	14.77		2.6		[42]	
CuO	0.1 M NaOH with different concentrations of glucose	168.7	0	2		10		[43]	
MOF derived CuO/C–400^o^C	Human blood sample	244.71	0.005	25.325		1		This work	

### Selectivity and stability of the resulting electrodes

**Fig. 5:**
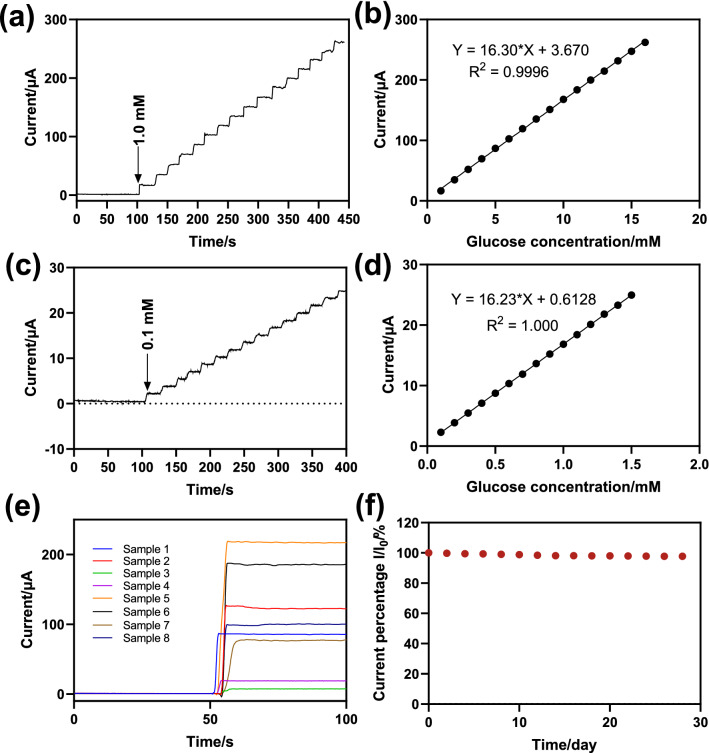
Amperometric responses of CuO/C-400 °C upon the consecutive addition of glucose in **a** artificial serum and **c** artificial saliva at 0.5 V (vs. Ag/AgCl); the corresponding calibration curves of the CuO/C-400 °C for the glucose detection in **b** artificial serum and **d** artificial saliva; **e** Amperometric i-t response of the CuO/C-400 °C electrodes at 0.5 V (vs. SCE) for different samples; **f** The relative current strength of the electrode within 28 days

It is considered that multiple possible interferents (NaCl, KCl, AA, fructose, and GSH) may coexist with glucose in a real serum environment. Figure 4c showed i-t curves of the sensor with successfully adding 1.0 mM glucose and 0.1 mM interferences in the electrolyte. The response current changed significantly after the addition of glucose. In contrast, the changed response currents were almost negligible when interfering substances were added to the electrolyte. Figure 4d showed the comparison of perturbation responsiveness and glucose responsiveness, and it can be clearly seen that the effect of perturbation was not obvious. Thus, the resulting electrodes demonstrated acceptable resistance to interference measurements. For the stability test, the sensors were stored in RT and tested every 2 days. As shown in Fig. 5f, the sensors still maintained good detection performance within 28 days, which was about 95% of the initial value, indicating that the obtained sensor can be harnessed to sensitively detect the glucose concentration after being placed for a long time.

### Real sample detection

As the CuO/C-400 °C nanoparticles containing oxygen vacancies with a remarkable ability to catalyze glucose oxidation and a good anti-interference ability. The prepared biosensor has also been explored with artificial serum/saliva (Fig. 5a–d) and real blood to establish its applicability in real-time detection. Specifically, the detection performance of the sensor in 0.1 M NaOH solution containing different biological samples was investigated by continuously adding an artificial serum and saliva containing 1 M and 0.1 M glucose, respectively, under an applied voltage of 0.5 V. The current response was found to increase with the increase of added artificial samples with perfect regression equations of I(µA) = 16.3 C(mM) + 3.670 µA (R^2^ = 0.9996) and I(µA) = 16.23 C(mM) + 0.6128 µA (R^2^ = 1.0000) from 1 to 16 mM and 0.1–1.5 mM, respectively.

In addition, the detection performance of the sensor was also verified by dropping different samples, including artificial and actual samples, directly on the surface without dilution (Fig. 5e). As shown in Table 2, the concentrations of the pieces were calculated referring to different equations mentioned above with high recovery from 97% to 103% in Table 2. In addition, the comparison of the glucose levels estimated with the fabricated biosensor and concentration recorded from the clinically available Hexokinase method was shown in Table 3. Herein, the designed CuO/C-400 °C sensor can be applied in different conditions with remarkable sensing performance. Notably, the screen-printed-based sensors can also be converted into portable detection platforms with miniature electrochemical workstations.

**Table 2:** Determination of glucose level in artificial samples

Sample	Added	Concentration (mM)	R.S.D. (%)	Recovery (%)
1	5.3	5.35	1.6	101
2	8.7	8.51	2.3	98
3	0.4	0.38	1.1	97
4	1.1	1.13	1.4	103

Sample 1–2: artificial serum, Sample 3–4: artificial saliva, *RSD* relative standard deviation for three independent measurements, Recovery: (detected /added ×100%)

**Table 3:** Determination of glucose level in clinical samples

Sample	Concentration recorded from hexokinase method (mM)	Concentration calculated from biosensor (mM)	R.S.D. (%)	Recovery (%)
5	13.2	12.81	2.2	97
6	11.7	11.82	1.8	101
7	4.7	4.61	2.3	98
8	6.1	5.92	2.1	97

Sample 5–6: two diabetics, Sample 7–8: two healthy persons, *RSD* relative standard deviation for three independent measurements, Recovery: (concentration calculated from our prepared sensor /concentration recoded from glucometer ×100%

## Conclusion

In this work, the CuO/C with the most abundant content of oxygen-vacancy was synthesized through pyrolyzing MOF as a sacrificial template at 400 °C with fast electron transfer, low overpotential, and high response current, which contributed to its excellent electrocatalytic activity toward glucose oxidation. The electrooxidation of glucose on the CuO/C-400 °C sensor was correlated with the pairing of vacancies (h^+^) and adsorbed OH^−^, resulting in high accumulated energy and oxidation of the glucose molecules. The sensing performance of CuO/C-400 °C was then applied with a wide linear range from 5.0 µM to 25.325 mM (R^2^ = 0.9998) and a good sensitivity of 244.71 µA mM^− 1^ cm^− 2^. In order to verify the accurate sample analysis, the CuO/C-400 °C sensor was also performed in simulated saliva/serum and human blood samples, and the results indicated that our prepared biosensor could effectively detect the glucose with high selectivity and recovery without further dilution, which opens up possibilities for ex vivo diagnostic applications for clinical and domestic use.

## Supplementary Information

**Additional file 1. **Experimental. **Figure S1.** **a** TEM and **b** element mapping of as-prepared Cu-MOF. **Figure S2.** XRD pattern of as-prepared Cu-MOF from 5^o^ to 80^o^. **Figure S3.** Survey scan of as-prepared Cu-MOF. **Figure S4.** High resolution XPS spectra of C_1s_,O_1s_ and Cu_2p_of the as-prepared Cu-MOF. **Figure S5.** TG curves and DTA of as-prepared Cu-MOFin the air. **Figure S6.** The SEM image of commercial CuO (inset: size distribution histogram). **Figure S7. **EDS result of CuO/C-400 ^o^C. **Figure S8.** XRD pattern of commercial CuO. **Figure S9. **Cyclic voltammograms of the **a** CuO/C-350 ^o^C, **b** CuO/C-450 ^o^C and **c** commercial CuO electrodes in 0.1 M NaOH with/without 0.2 mM glucose at a scan rate of 100 mV s^−1^. **Figure S10.** CV curves of **a** CuO/C-350 ^o^C and **b** CuO/C-450 ^o^C in 0.5 mM K_3_Fe(CN)_6_/0.1 M KCl electrolyte at different scan rate and **c**, **d** the corresponding fitting curves. **Figure S11. **Amperometric i-t response of the CuO/C-400 ^o^Celectrodes in 0.1 M NaOH at different voltage (vs. SCE) with stirring. **Figure S12. a** Amperometric responses of CuO/C-350 ^o^C in 0.1 M NaOH upon consecutive addition of glucose at 0.5 V (vs. Ag/AgCl) and **b** correspondingcalibration curves of the CuO/C-350 ^o^C for glucose detection. **Figure S13. a** Amperometric responses of CuO/C-450 ^o^C in 0.1 M NaOH upon consecutive addition of glucose at 0.5 V (vs. Ag/AgCl) and **b** the corresponding calibration curves of CuO/C-450 ^o^C for glucose detection._._
