# Supplementary material for: MOF derived core-shell CuO/C with temperature-controlled oxygen-vacancy for real time analysis of glucose
Source: J Nanobiotechnology. 2022 Dec 1;20:507. doi: 10.1186/s12951-022-01715-z (PMC9714170; doi:10.1186/s12951-022-01715-z)
Supplement: Supplementary file 1 — Additional file 1. Experimental. Figure S1. a TEM and b element mapping of as-prepared Cu-MOF. Figure S2. XRD pattern of as-prepared Cu-MOF from 5o to 80o. Figure S3. Survey scan of as-prepared Cu-MOF. Figure S4. High resolution XPS spectra of C1s,O1s and Cu2pof the as-prepared Cu-MOF. Figure S5. TG curves and DTA of as-prepared Cu-MOFin the air. Figure S6. The SEM image of commercial CuO (inset: size distribution histogram). Figure S7. EDS result of CuO/C-400 oC. Figure S8. XRD pattern of commercial CuO. Figure S9. Cyclic voltammograms of the a CuO/C-350 oC, b CuO/C-450 oC and c commercial CuO electrodes in 0.1 M NaOH with/without 0.2 mM glucose at a scan rate of 100 mV s−1. Figure S10. CV curves of a CuO/C-350 oC and b CuO/C-450 oC in 0.5 mM K3Fe(CN)6/0.1 M KCl electrolyte at different scan rate and c, d the corresponding fitting curves. Figure S11. Amperometric i-t response of the CuO/C-400 oCelectrodes in 0.1 M NaOH at different voltage (vs. SCE) with stirring. Figure S12. a Amperometric responses of CuO/C-350 oC in 0.1 M NaOH upon consecutive addition of glucose at 0.5 V (vs. Ag/AgCl) and b correspondingcalibration curves of the CuO/C-350 oC for glucose detection. Figure S13. a Amperometric responses of CuO/C-450 oC in 0.1 M NaOH upon consecutive addition of glucose at 0.5 V (vs. Ag/AgCl) and b the corresponding calibration curves of CuO/C-450 oC for glucose detection.. [file 12951_2022_1715_MOESM1_ESM.docx]

**MOF derived core-shell CuO/C with temperature-controlled oxygen-vacancy for real time analysis of glucose**

Chen Zhao^1,2^, Xiaoying Tang^2^, Jinge Zhao^3^, Jie Cao^3,*^, Zhenqi Jiang^2,*^ Jieling Qin^1,*^

1. Tongji University Cancer Center, Shanghai Tenth People's Hospital, School of Medicine, Tongji University, Shanghai 200092, China

2. School of Medical Technology, School of Life Science, Beijing Institute of Technology, Beijing 100081, China

3. Key Laboratory of Medical Molecule Science and Pharmaceutics Engineering, Ministry of Industry and Information Technology, Key Laboratory of Cluster Science of Ministry of Education, Beijing Key laboratory of Photoelectronic/Electro-photonic Conversion Materials, School of Chemistry and Chemical Engineering, Beijing Institute of Technology, Beijing 100081, P. R. China.

* Corresponding Author

Email: qinjieling770@hotmail.com (Jieling Qin)

7520200073@bit.edu.cn (Zhenqi Jiang)

[jcao@bit.edu.cn (Jie](mailto:jcao@bit.edu.cn%20(Jie) Cao)

Additional file 1: **Experimental**

**Materials and regents**

Cu(NO_3_)_2_·6H_2_O, 1,3,5-Benzenetricarboxylic acid, ethanol, commercial CuO (40 nm) and triethylamine was bought from Adamas-beta® (Shanghai, China). NaCl, KCl, Glu, NaOH, Ascorbic Acid, GSH, K_3_[Fe(CN)_6_] and maltose Aladdin (Shanghai, China). The ultrapure water (18.2 MΩ cm^−1^) used in this study came from a Milli-Q ultrapure system.

**Characterization**

Scan electron microscopy (SEM) was detected on JOEL JSM-7500F (JEOL, Japan) at 10 kV. Transmission electron microscopy (TEM), High-resolution transmission electron microscopy (HRTEM) images and Energy Dispersive Spectrometer (EDS) mapping were detected on a JEOL2100 (JEOL, Japan) at 120 kV. The structural properties of samples were investigated by X-ray diffraction (XRD) using an X-ray powder diffractometer (XRD, D8 Discover, Bruker AXS). The TG-DTA was tested by DTG-60H (Shimadzu, Japan). The element state was detected by X-ray photoelectron spectroscopy (XPS) on X-ray photoelectron spectroscopy (PHI VersaProbe III, Ulvac-Phi, Japan). All electrochemical experiments were performed using a model CHI760E electrochemical workstation (Shanghai Chenhua Instruments, Shanghai, China) and the data were recorded with corresponding software. A conventional three-electrode system was employed with a modified GCE electrode, an Ag/AgCl electrode, and a Pt wire as the working, reference, and counter electrodes, respectively. The cyclic voltammetry (CV) tests were performed at a scan rate of 100 mV/s with the potential range of -0.2 to 0.7 V vs. Ag/AgCl. Amperometric i-t curve tests were performed at 0.5 V in 50 ml of 0.1 M NaOH aqueous solution under stirring. The electron transfer resistance in 50 mL of 0.1M KCl solution with 5 mM Fe(CN)_6_^3-/4-^ was assessed by the electrochemical impedance spectroscopy (EIS). All the measurements were performed at RT.

**Hexokinase method detection of glucose in human plasma**

3 μL plasma samples were taken and added 240 μL reagents 1 (1.4 mmol/L adenosine triphosphate, 0.8 mmol/L oxidized coenzyme I, 3800 U/L glucose-6-phosphate dehydrogenase, 50 mmol/L triethanolamine buffer); 60 μL reagents 2 (2500 U/L hexokinase, 50 mmol/L triethanolamine buffer). After 300 s reaction time, the absorbance increase rate was monitored at the wavelength of 340 nm and the glucose concentration in the plasma was calculated.


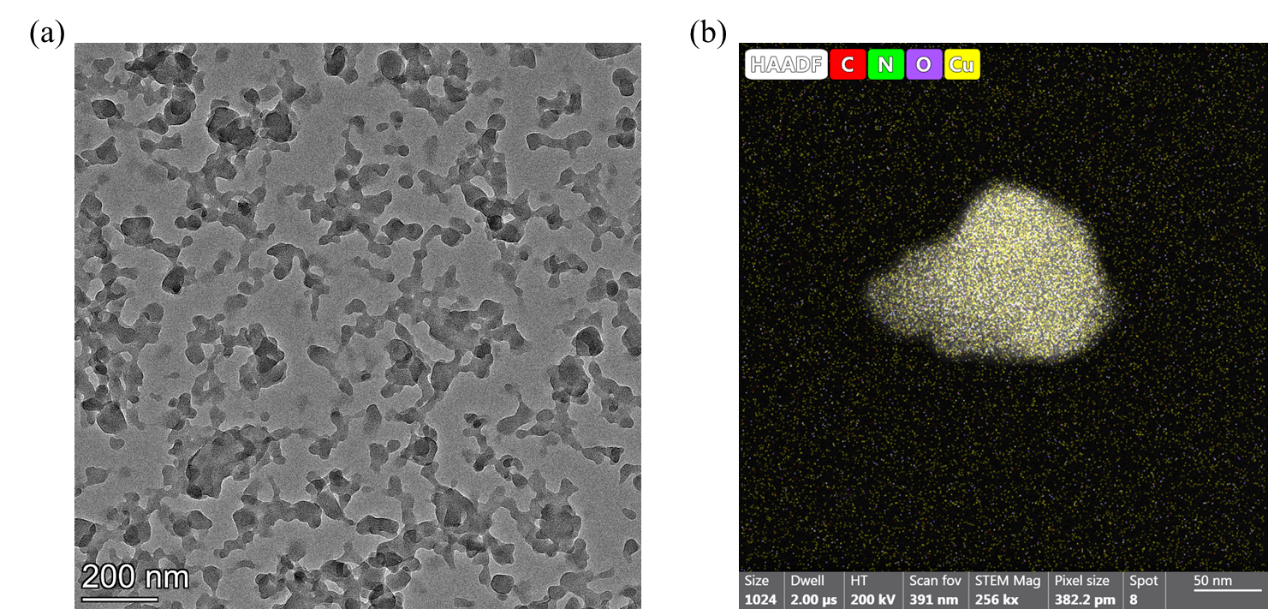


**Figure S1.** (a) TEM and (b) element mapping of as-prepared Cu-MOF.


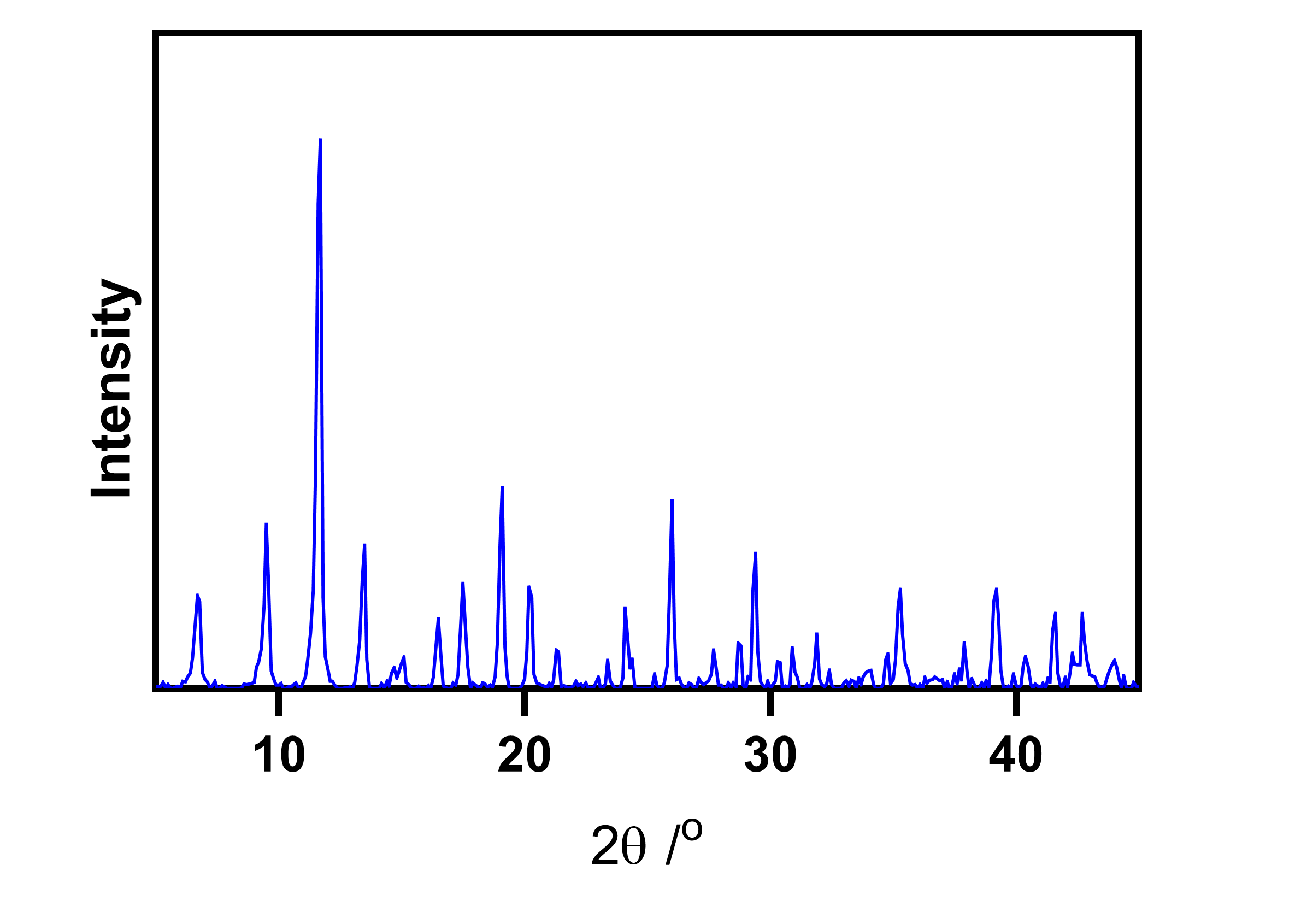


**Figure S2.** XRD pattern of as-prepared Cu-MOF from 5^o^ to 80^o^.


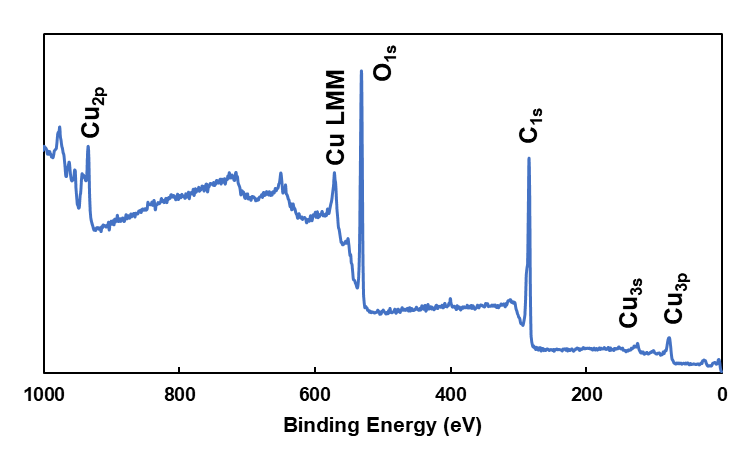


**Figure S3.** Survey scan of as-prepared Cu-MOF.


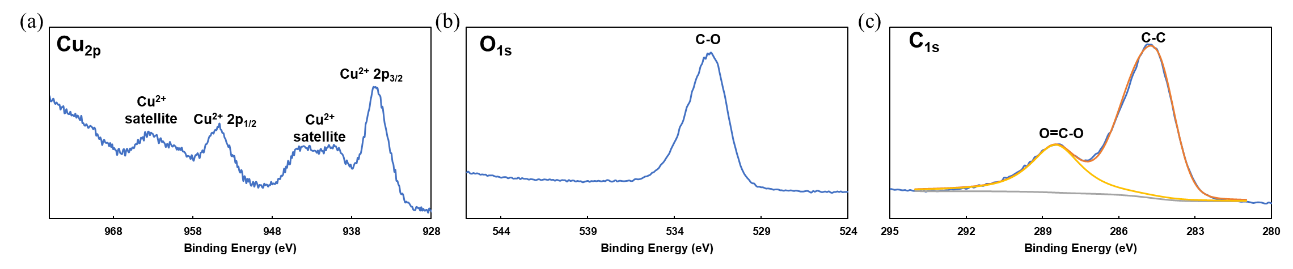


**Figure S4.** High resolution XPS spectra of C_1s_, O_1s_ and Cu_2p_ of the as-prepared Cu-MOF_._


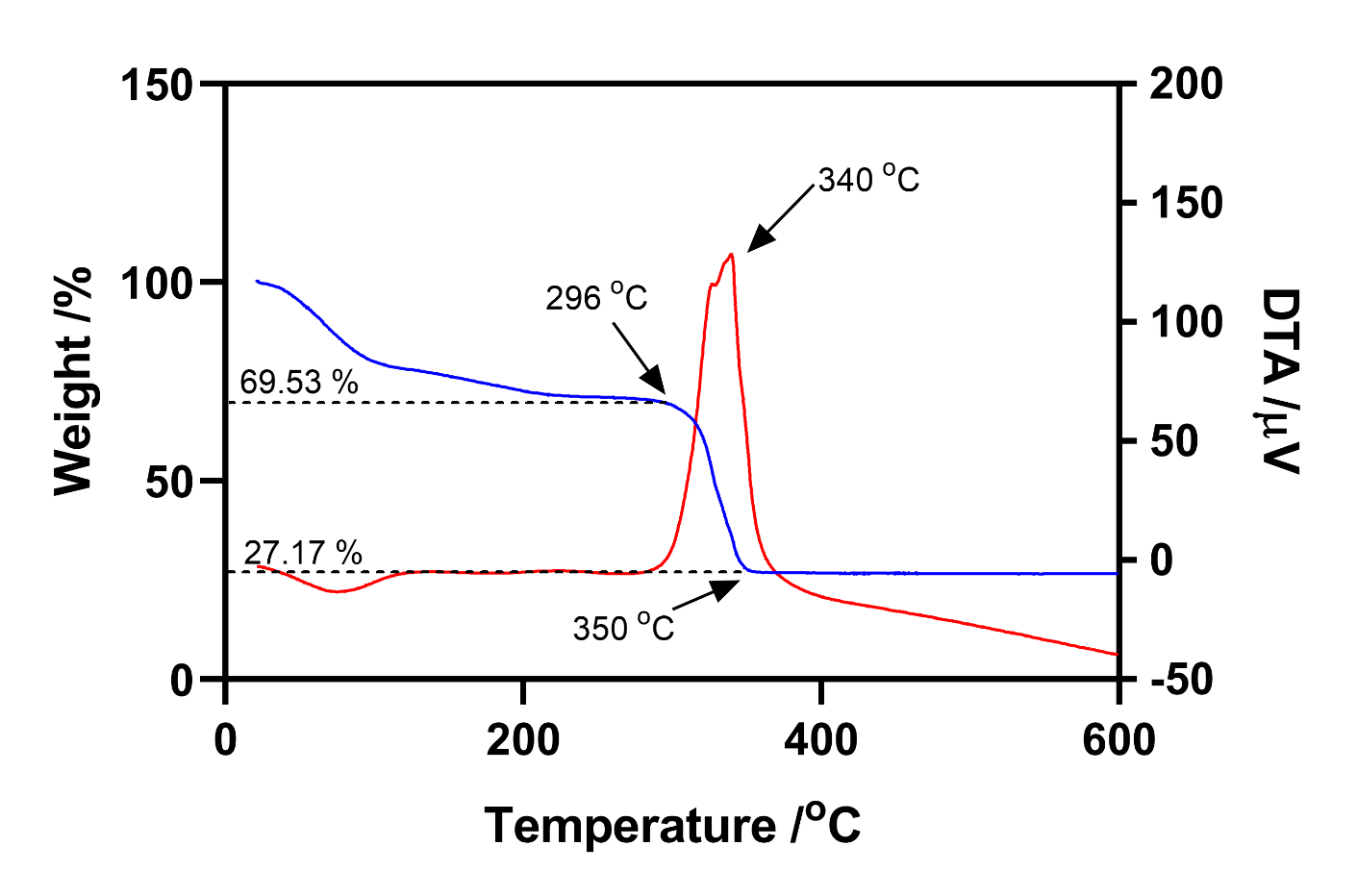


**Figure S5.** TG curves and DTA of as-prepared Cu-MOF in the air;


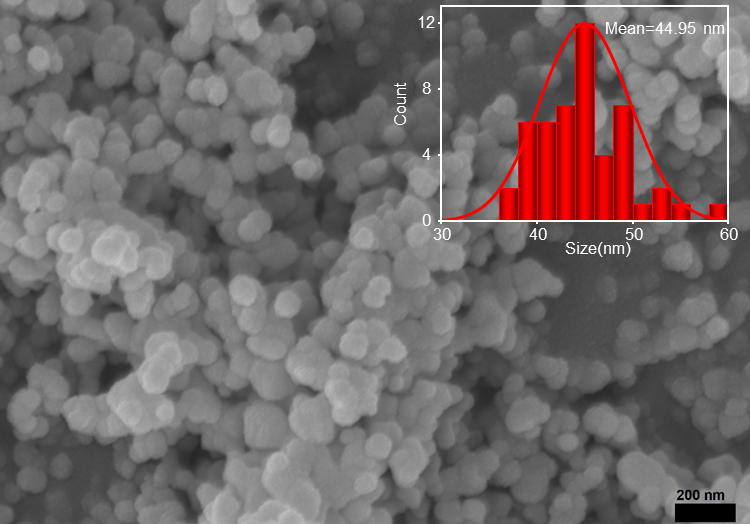


**Figure S6.** The SEM image of commercial CuO (inset: size distribution histogram).


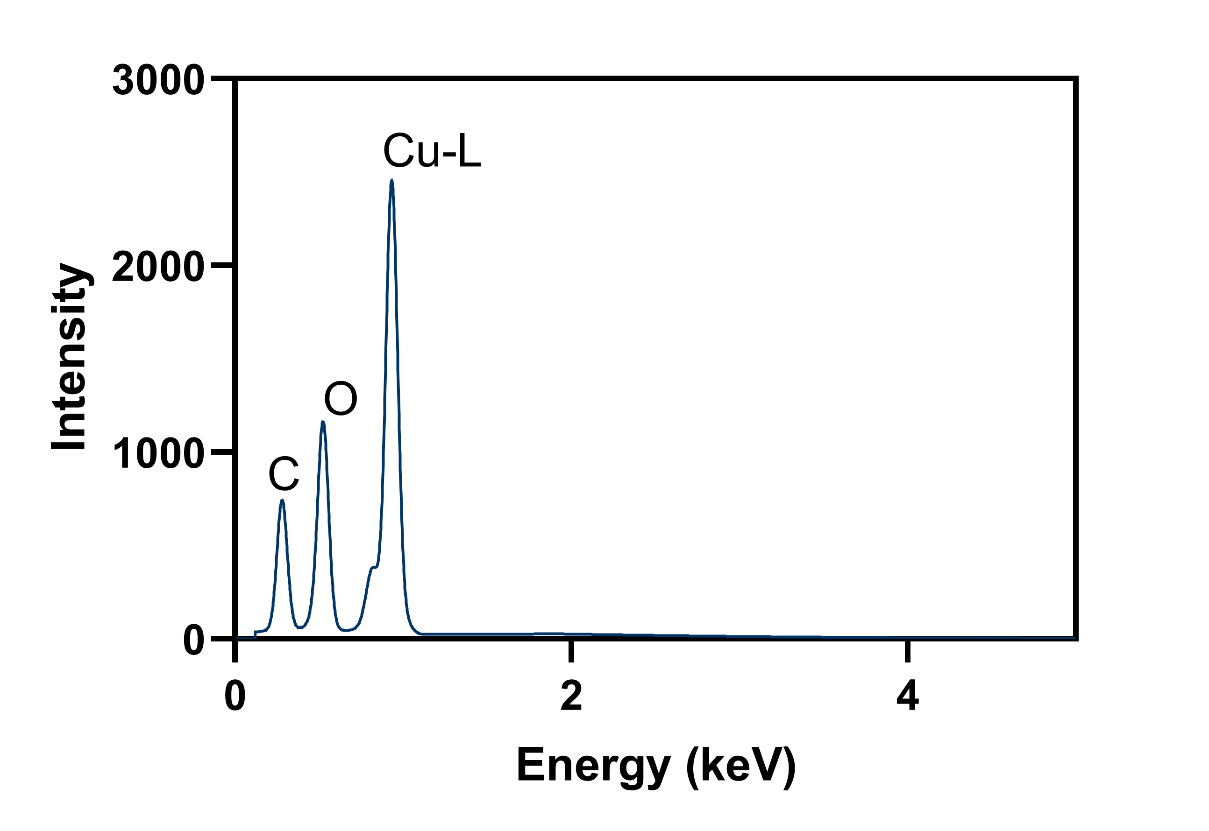


**Figure S7.** EDS result of CuO/C-400^o^C.


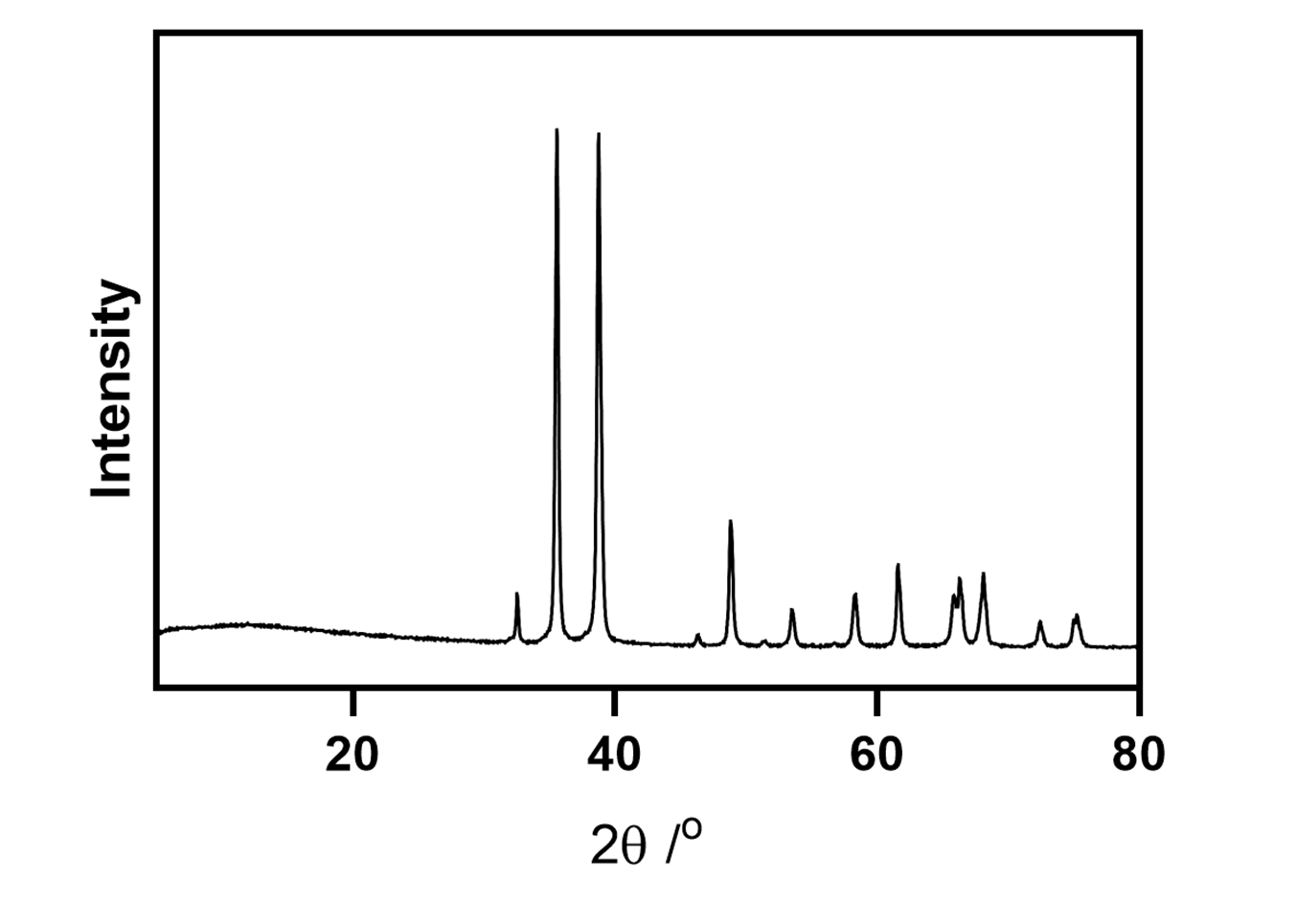


**Figure S8.** XRD pattern of commercial CuO.


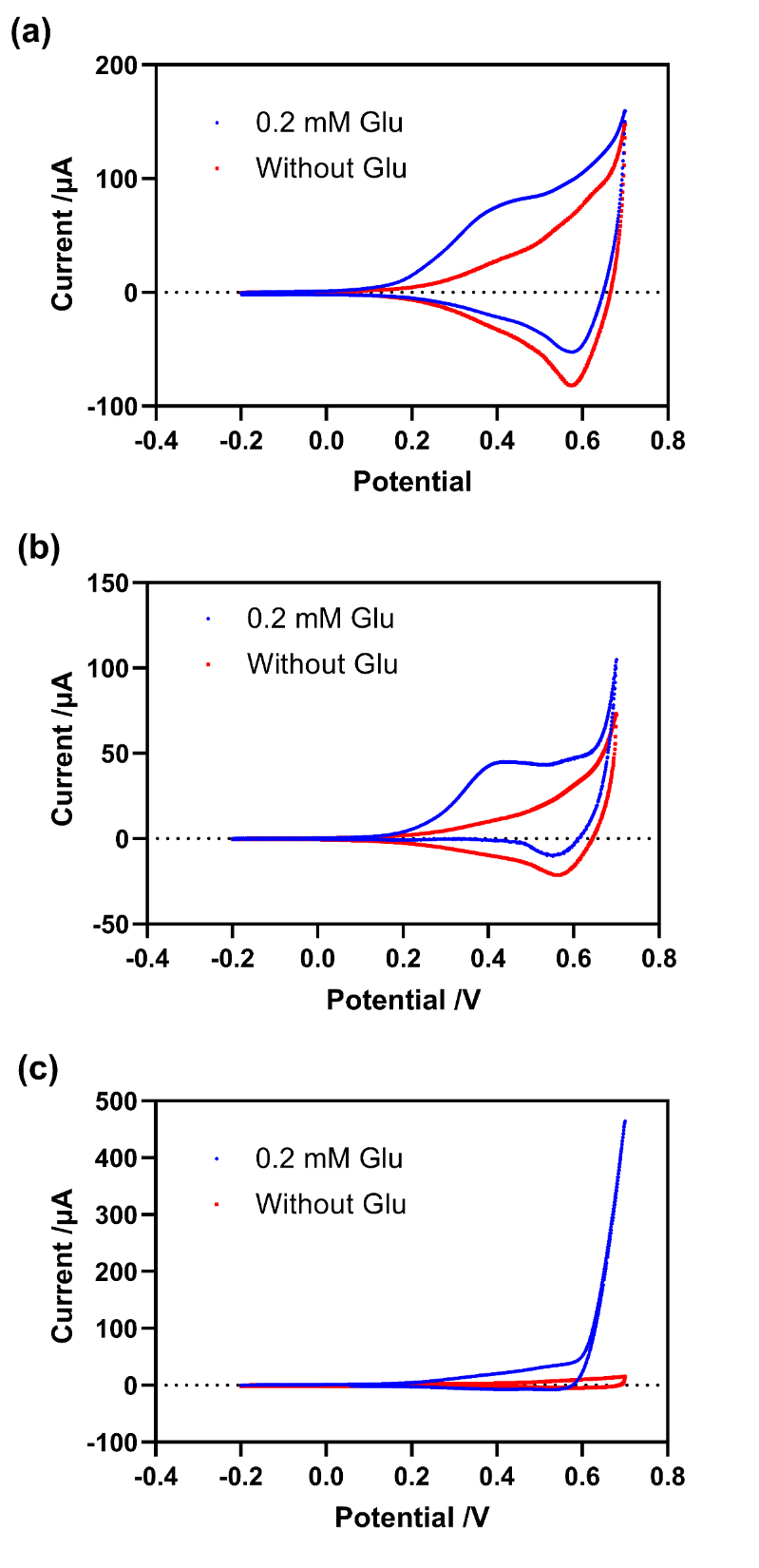


**Figure S9.** Cyclic voltammograms of the (a) CuO/C-350^o^C, (b) CuO/C-450^o^C and (c) commercial CuO electrodes in 0.1 M NaOH with/without 0.2 mM glucose at a scan rate of 100 mV s^−1^.


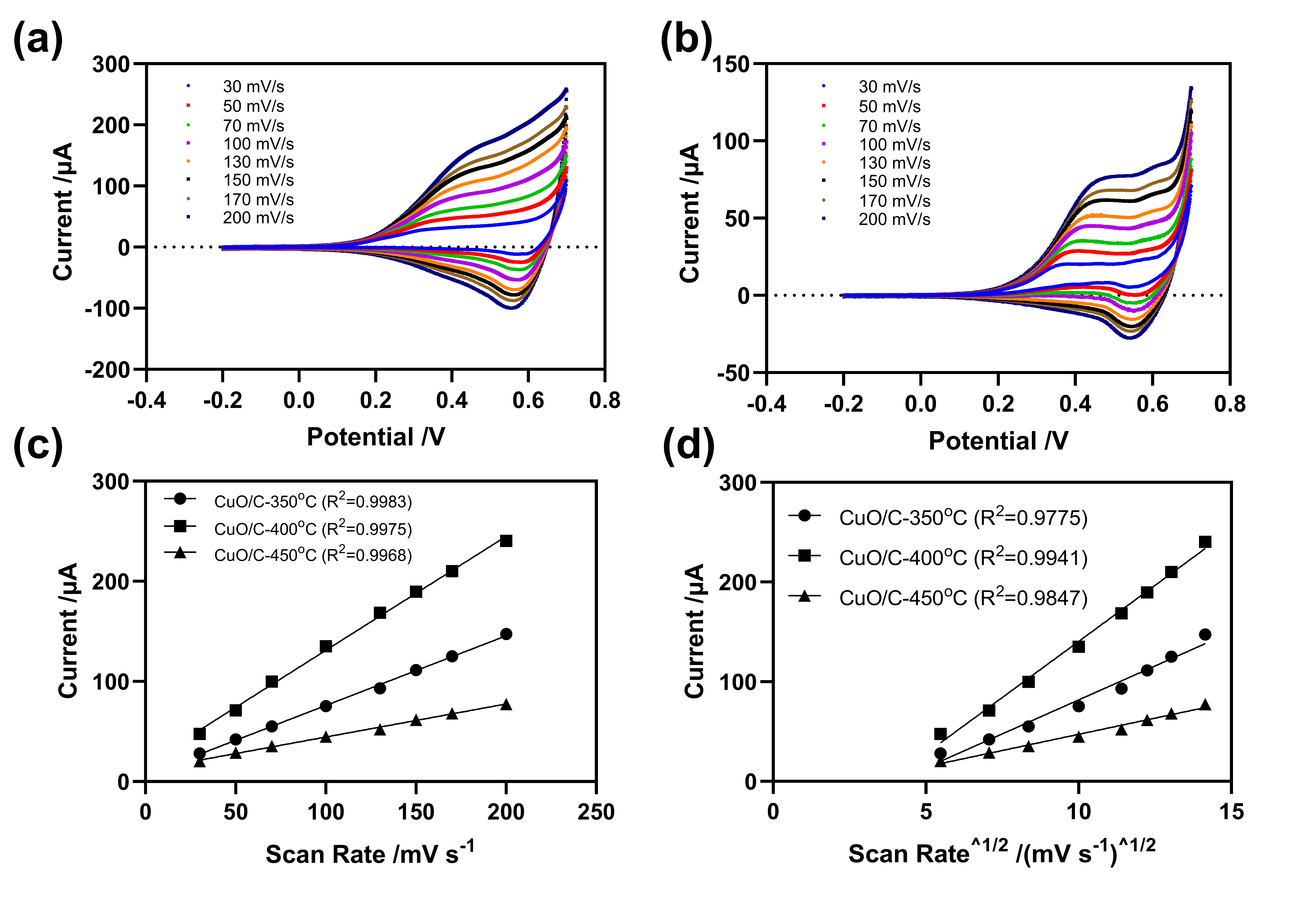


**Figure S10.** CV curves of (a) CuO/C-350^o^C and (b) CuO/C-450^o^C in 0.5 mM K_3_Fe(CN)_6_/0.1 M KCl electrolyte at different scan rate and (c, d) the corresponding fitting curves.


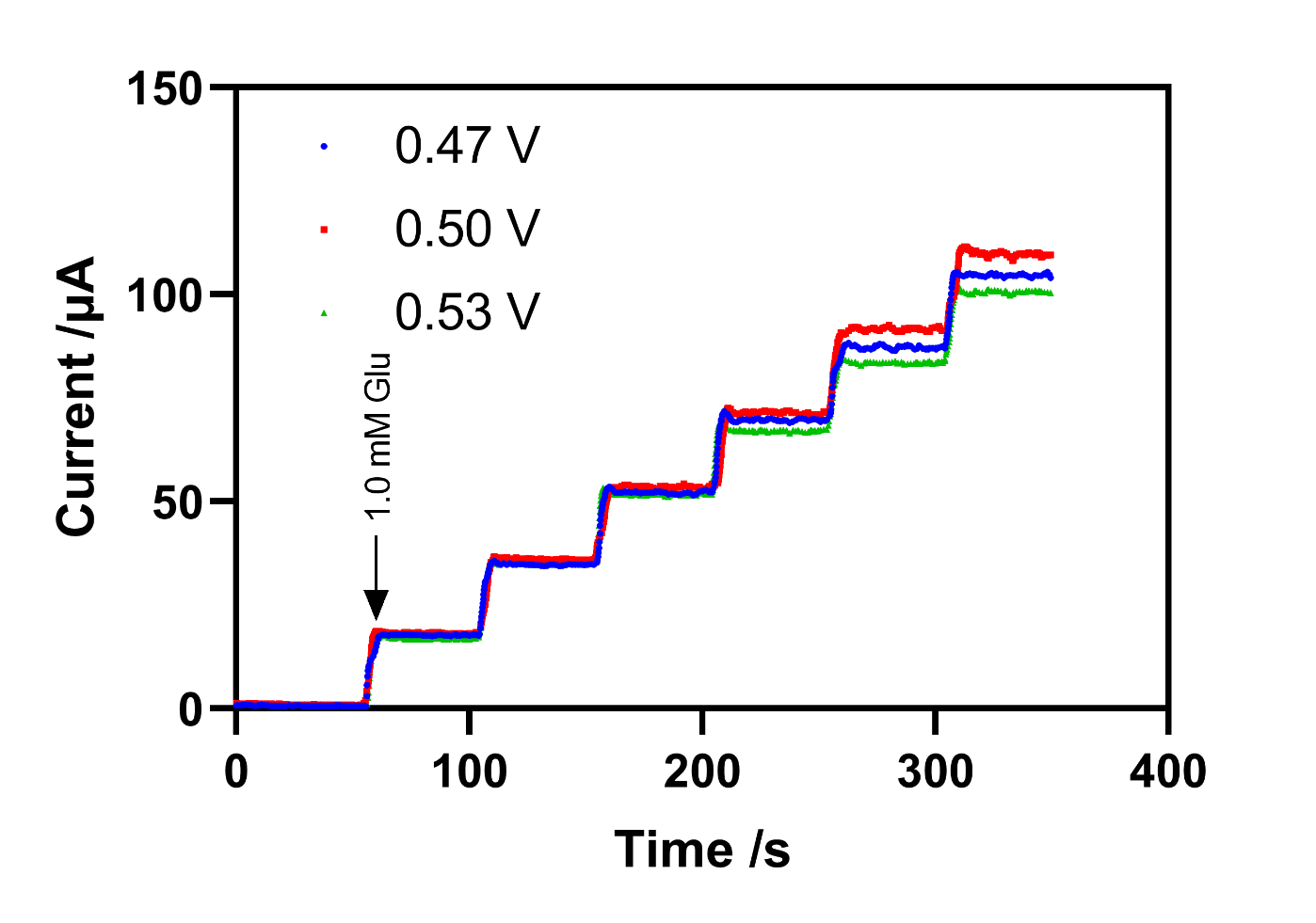


**Figure S11.** Amperometric i-t response of the CuO/C-400^o^C electrodes in 0.1 M NaOH at different voltage (vs. SCE) with stirring;


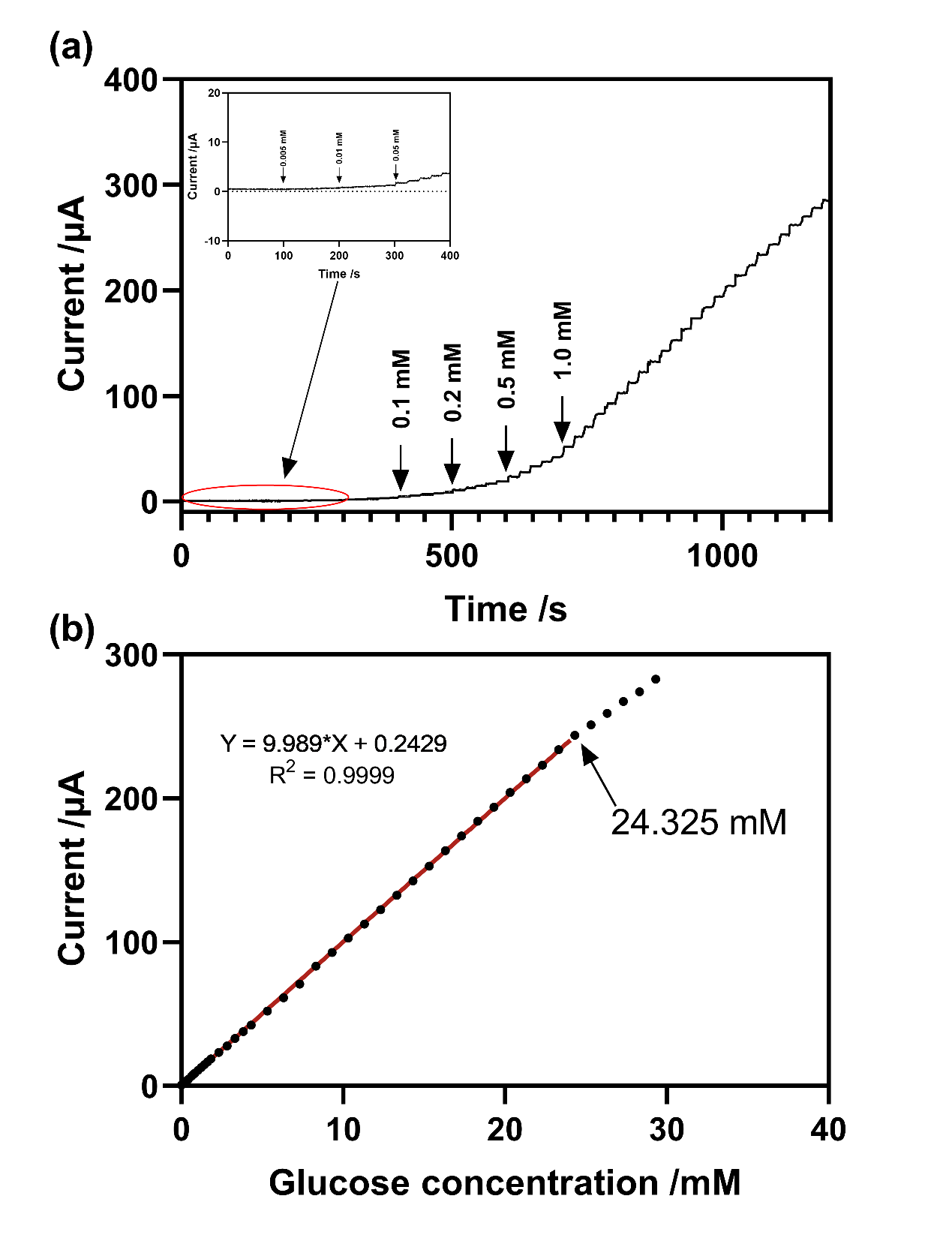


**Figure S12.** (a) Amperometric responses of CuO/C-350 ^o^C in 0.1 M NaOH upon consecutive addition of glucose at 0.5 V (vs. Ag/AgCl) and (b) corresponding calibration curves of the CuO/C-350 ^o^C for glucose detection.


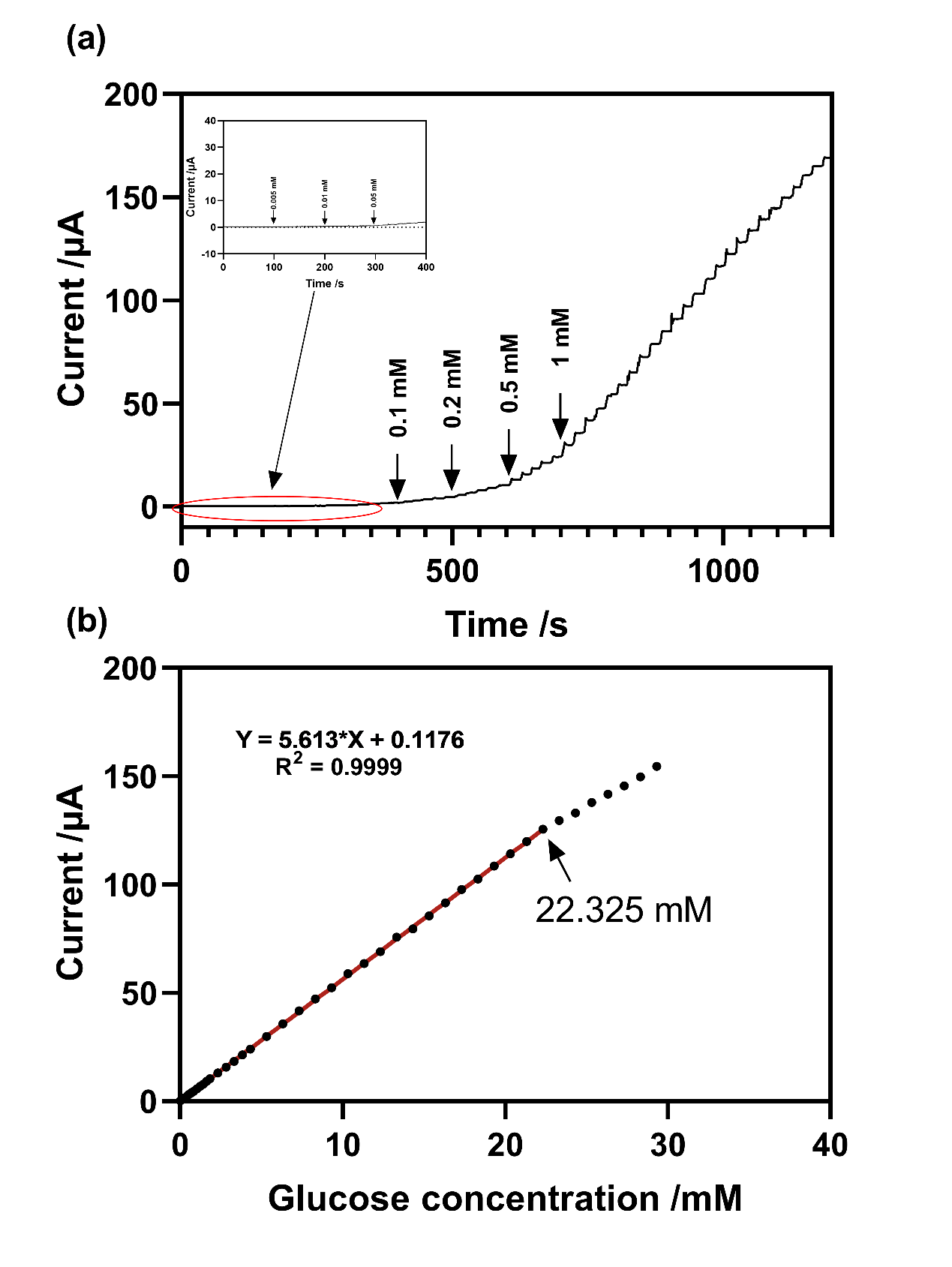


**Figure S13.** (a) Amperometric responses of CuO/C-450^o^C in 0.1 M NaOH upon consecutive addition of glucose at 0.5 V (vs. Ag/AgCl) and (b) the corresponding calibration curves of CuO/C-450 ^o^C for glucose detection.
